# Geography and Environment Shape Landscape Genetics of Mediterranean Alpine Species *Silene ciliata* Poiret. (Caryophyllaceae)

**DOI:** 10.3389/fpls.2018.01698

**Published:** 2018-11-27

**Authors:** Javier Morente-López, Cristina García, Carlos Lara-Romero, Alfredo García-Fernández, David Draper, José María Iriondo

**Affiliations:** ^1^Área de Biodiversidad y Conservación, Escuela Superior de Ciencias Experimentales y Tecnología (ESCET), Universidad Rey Juan Carlos, Madrid, Spain; ^2^Department of Evolution, Ecology and Behaviour, Institute of Integrative Biology, University of Liverpool, Liverpool, United Kingdom; ^3^Plant Biology Group, CIBIO/InBio, Centro de Investigação em Biodiversidade e Recursos Genéticos, Laboratório Associado, Universidade do Porto, Porto, Portugal; ^4^Global Change Research Group, Mediterranean Institute for Advanced Studies (IMEDEA), Consejo Superior de Investigaciones Científicas (CSIC), Esporles, Spain; ^5^Natural History and Systematics Research Group, cE3c, Centro de Ecologia, Evolução e Alterações Ambientais, Universidade de Lisboa, Lisbon, Portugal; ^6^UBC Botanical Garden and Centre for Plant Research, Department of Botany, The University of British Columbia, Vancouver, BC, Canada

**Keywords:** landscape genetics, isolation by distance, isolation by resistance, isolation by environment, genetic diversity, marginal populations, environmental gradient

## Abstract

The study of the drivers that shape spatial genetic structure across heterogeneous landscapes is one of the main approaches used to understand population dynamics and responses in changing environments. While the Isolation-by-Distance model (IBD) assumes that genetic differentiation increases among populations with geographical distance, the Isolation-by-Resistance model (IBR) also considers geographical barriers and other landscape features that impede gene flow. On the other hand, the Isolation-by-Environment model (IBE) explains genetic differentiation through environmental differences between populations. Although spatial genetic studies have increased significantly in recent years, plants from alpine ecosystems are highly underrepresented, even though they are great suitable systems to disentangle the role of the different factors that structure genetic variation across environmental gradients. Here, we studied the spatial genetic structure of the Mediterranean alpine specialist *Silene ciliata* across its southernmost distribution limit. We sampled three populations across an altitudinal gradient from 1850 to 2400 m, and we replicated this sample over three mountain ranges aligned across an E-W axis in the central part of the Iberian Peninsula. We genotyped 20 individuals per population based on eight microsatellite markers and used different landscape genetic tools to infer the role of topographic and environmental factors in shaping observed patterns along the altitudinal gradient. We found a significant genetic structure among the studied *Silene ciliata* populations which was related to the orography and E-W configuration of the mountain ranges. IBD pattern arose as the main factor shaping population genetic differentiation. Geographical barriers between mountain ranges also affected the spatial genetic structure (IBR pattern). Although environmental variables had a significant effect on population genetic diversity parameters, no IBE pattern was found on genetic structure. Our study reveals that IBD was the driver that best explained the genetic structure, whereas environmental factors also played a role in determining genetic diversity values of this dominant plant of Mediterranean alpine environments.

## Introduction

Species resilience to changing environments largely depends on their genetic diversity (Lande and Shannon, 1996; Frankham et al., 2002). Thus, knowledge on the spatial distribution of species genetic diversity is an essential component for understanding the challenges brought about by climate change (Manel and Holderegger, 2013). This is especially important for species in habitats with marked environmental gradients, such as those found in mountain ecosystems. Projected climate warming rates are expected to have a great impact on mountain ecosystems (Beniston et al., 1997; Nogués-Bravo et al., 2007; Kohler et al., 2010; Rangwala and Miller, 2012), particularly on alpine plant communities (Thuiller et al., 2005; Gottfried et al., 2012; Steinbauer et al., 2018). It is, therefore, crucial to identify the main ecological drivers of genetic structure at different spatial scales to forecast the response of alpine plant species to climate change.

Several hypotheses have been proposed to generalize patterns of genetic variation across space. Gene flow limitation and drift caused by geographical isolation generate an Isolation-by-Distance (IBD) pattern (Wright, 1943). It assumes that linear relationship between genetic and Euclidean geographic distance *per se* is the main driver of genetic structure and considers that other factors like landscape features, range boundaries or environmental characteristics influencing gene flow are not relevant (Manel et al., 2003; McRae, 2006; Storfer et al., 2007). Nowadays, there is evidence that IBD may be too simplistic and that it should be contrasted with more complex models (Jenkins et al., 2010). Thus, the Isolation-by-Resistance (IBR) pattern incorporates range boundaries and landscape features, such as geographical barriers, as a cause of gene flow limitation and drift (McRae, 2006; Spear et al., 2010; Cushman et al., 2015). Both IBD and IBR are closely related to the underlying process of Isolation-by-Dispersal and indirectly related to environmental conditions (Orsini et al., 2013). Isolation-by-Environment (IBE) pattern is caused by environmental heterogeneity and local adaptation related to strong divergent selection (Sexton et al., 2014; Wang and Bradburd, 2014). In mountain ecosystems, high isolation produced by steep topography, barriers and environmental gradients facilitate various processes that generate different isolation patterns. Historical patterns of range expansion and contraction can also modify current isolation effects and affect genetic structure (Cushman et al., 2015). All these processes can act alone or in combination, and their importance may vary depending on the spatial scale of observation (Orsini et al., 2013).

Isolation processes related to geographic and environmental distances may produce profound demographic and genetic outcomes for plant populations within species distribution ranges (Eckert et al., 2008). Species range limits, characterized by increased genetic isolation (Sexton et al., 2009), often occur across ecological gradients where habitats become less suitable and environmental differentiation increases when moving toward the margins (Kawecki, 2008). Inside species distribution ranges, we can distinguish populations inhabiting environmentally central areas from populations occurring in marginal areas, i.e., those located at the edge of the species environmental and/or geographical distribution range (Soule, 1973; Brussard, 1984; Kawecki, 2008; Pironon et al., 2015; Papuga et al., 2018). It is important to consider that the geographical center of the species range is not necessarily associated to the areas with higher habitat quality (Sagarin et al., 2006). In the distribution margin, populations experience environmental conditions that differ from those found at the central areas and they tend to be prone to environmental fluctuations that ultimately reduce habitat quality and quantity and restrict resource availability, affecting population demography and thus genetic diversity (Hampe and Petit, 2005; Eckert et al., 2008; Kawecki, 2008). Thus, as we approach to the distribution margins it is expected that other processes such as IBE will influence the distribution of genetic diversity, in addition to IBD.

Plants from alpine ecosystems provide a very interesting context to disentangle the various mechanisms that structure genetic variation within and among populations at different scales related to patterns of isolation between populations (IBD/IBR vs. IBE). Several studies have shown adaptive genetic differentiation and local adaptation processes related with the strong environmental variability in these ecosystems using phenotypic, genetic, and/or genomic data (e.g., Gonzalo-Turpin and Hazard, 2009; Frei et al., 2012a, 2014; Di Pierro et al., 2017; Hämälä et al., 2018). In some instances, local adaptation persists despite the existence of significant gene flow between populations (Gonzalo-Turpin and Hazard, 2009; Kim and Donohue, 2013). However, in other cases, local adaptation has not been found (Ometto et al., 2015; Hirst et al., 2016; Hamann et al., 2017). Also the effect of geographical distance and landscape configuration have been documented as an important forces that hinder gene flow and promote genetic differentiation between populations (Kuss et al., 2008; Aægisdóttir et al., 2009; Geng et al., 2009; Wang et al., 2011; De Vriendt et al., 2017). However, few studies have combined different isolation processes in the context of landscape genetic approaches (e.g., Mosca et al., 2014; Wu et al., 2015; Noguerales et al., 2016) and focused on environmental variables causing the genetic isolation patters identification (e.g., Manel et al., 2012; Mosca et al., 2012). Furthermore, although landscape genetics studies have increased significantly in recent years, plants are still highly underrepresented (Storfer et al., 2010; DiLeo and Wagner, 2016).

Mediterranean mountain ranges encompass a territory with wide environmental and geographical heterogeneity where many species reach their southernmost distribution range limits. They are also rich in endemic species and are biogeographically important as glacier refuges (Nieto Feliner, 2014). In this context, we combined geographic, environmental and genetic data and applied landscape genetics tools to depict the genetic structure patterns of nine populations of a Mediterranean alpine plant, *Silene ciliata* Poiret *(Caryophyllaceae)*, distributed along the elevational range of the species in central Spain. As an alpine species, the distribution range of *S. ciliata* has experienced historical environmental fluctuations related to glaciations (Hewitt, 2001; Nieto Feliner, 2011) and is presently challenged by environmental changes related to climate change (Körner, 2003, 2007). The species grows across an elevational gradient, where populations at the lowest elevation experience the most stressful conditions (due to water limitation in summer), constraining seedling establishment and reproductive performance (Giménez-Benavides et al., 2007a, 2011b; Lara-Romero et al., 2014). Our aim was to assess the role of geographic isolation (IBD), landscape features (IBR) and environmental heterogeneity (IBE) in shaping the genetic diversity and structure of *S. ciliata*. We specifically addressed three main questions: (i) Are *S. ciliata* populations genetically structured across mountain ranges and elevational gradients? (ii) If so, can observed genetic structure patterns be explained by isolation-by-distance (IBD), isolation by resistance (IBR) and/or isolation-by-environment (IBE)? and (iii) How do these factors affect genetic diversity?

Most genetic approaches are addressed to species with limited distribution ranges, whereas more widespread species have not deserved so much attention from the conservation genetics community. The results of this study will provide useful information for the conservation of the genetic variation of a dominant species of the endangered Mediterranean alpine ecosystems (Tutin et al., 1964; Kyrkou et al., 2015) in one of its southernmost distribution limits.

## Materials and Methods

### Study Species and Sampling Design

**FIGURE 1:**
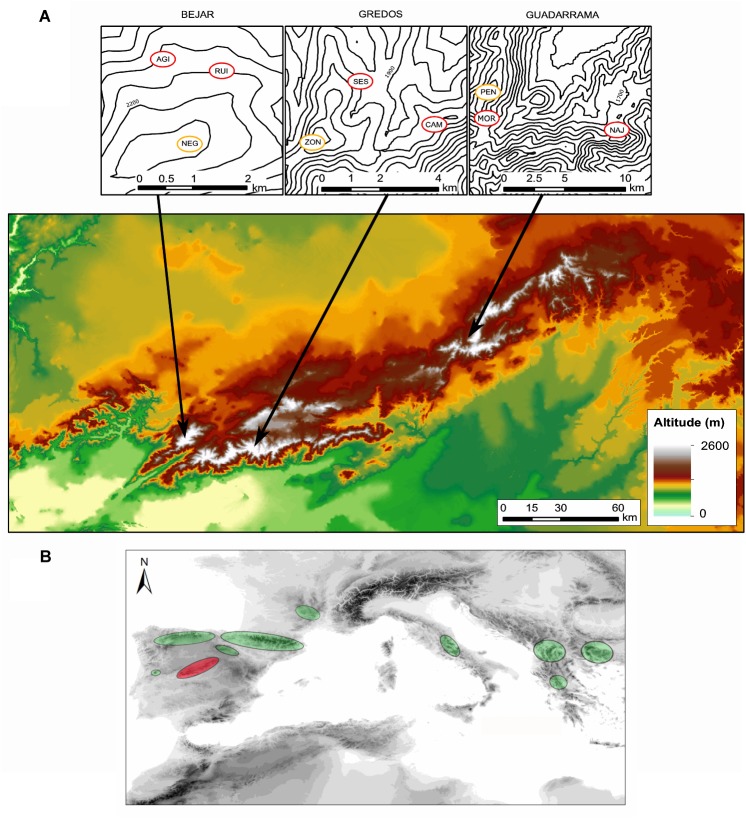
**(A)** Location of the study sites in the Sistema Central of the Iberian Peninsula. Yellow circles represent each of the three populations located in optimal areas and red dots represent each of the six populations located in marginal areas (see Table 1). NAJ, Najarra Baja; MOR, Morrena Peñalara; PEN, Pico Peñalara; SES, El Sestil; CAM, Los Campanarios; ZON, Altos del Morezón; RUI, Las Cimeras; AGI, Pico del Aguila; NEG, Canchal Negro. **(B)** Representation of *Silene ciliata* distribution area. Red circle indicates the Sistema Central of the Iberian Peninsula, where our study takes place.

*Silene ciliata* Poiret (Caryophyllaceae) is a dwarf cushion perennial plant which inhabits Mediterranean alpine habitats with marked environmental gradients. It is pollinated by nocturnal insects, mainly belonging to the genus *Hadena* (Lepidoptera, Noctuidae), and diurnal insects (Giménez-Benavides et al., 2007b). The flowering period spans from the end of July to the end of August. Seeds are relatively small (mean ± SD: 1.53 ± 0.49 mm diameter, 0.59 ± 0.06 mg weight), and most of them are dormant and need cold stratification to germinate (Giménez-Benavides et al., 2005; García-Fernández et al., 2015). The species is essentially barochorous, i.e., seeds lack any specific structure to promote dispersal. Thus, effective seed dispersal distances are low and relatively invariant across populations (mean ± SE: 0.40 ± 0.08 m, Lara-Romero et al., 2014). The distribution range of *S. ciliata* comprises the mountain ranges of the Northern Mediterranean area from Spain to Bulgaria (Tutin et al., 1964; Kyrkou et al., 2015), reaching its southernmost limit in the Sistema Central of the Iberian Peninsula (Figure 1). *S. ciliata* populations from the Sistema Central have the same phylogenetic origin as shown by chloroplast DNA analysis (Kyrkou et al., 2015). In these areas the species grows from 1900 to 2590 m in dry cryophilic pastures above the tree line. This Mediterranean Alpine ecosystem presents a pronounced summer drought combined with high solar radiation which induces typical xerophilic characteristics in the inhabiting species (Rivas-Martínez et al., 1990). The study took place in three mountain ranges of the Sistema Central (Spain): Guadarrama (GDM), Béjar (BJR) and Gredos (GRD) (Figure 1 and Table 1). The Sistema Central is a southwest-northeast oriented mountain range of approximately 500 km located in the center of the Iberian Peninsula. The GDM, GRD and BJR mountain ranges are located in the western, central and eastern areas of the Sistema Central, respectively.

**Table 1:** Geographic and environmental features of nine sampled populations of *Silene ciliata*. Env. Class., populations environmental classification; Tmax, Annual maximum temperature; Tmin, annual minimum temperature.

Population	Pop ID	Mountain range	Env. Class.	Elevation (m)	Tmax (°C)	Tmin (°C)	Lat.	Long.
Najarra baja	NAJ	Guadarrama (GDM)	Marginal	1850	26,5	-5,9	40°49′23,46″N	3°49′52.53″W
Morrena Peñalara	MOR	Guadarrama	Marginal	1980	24.7	-5.7	40°50′11.82″N	3°57′0.91″W
Pico de Peñalara	PEN	Guadarrama	Optimal	2400	24.1	-7.8	40°51′2.11″N	3°57′24.02″W
El Sestil	SES	Gredos (GRD)	Marginal	1900	28	-5.9	40°16′24.45″N	5°14′54.93″W
Los Campanarios	CAM	Gredos	Marginal	2000	27.7	-6	40°15′42.63″N	5°12′55.74″W
Altos del Morezón	ZON	Gredos	Optimal	2380	26.9	-7.7	40°14′57.5″N	5°16′8.3″W
Las Cimeras	RUI	Bejar (BJR)	Marginal	2000	26.7	-6.7	40°21′7.03″N	5°40′59.71″W
Pico El Aguila	AGI	Bejar	Marginal	1950	26.9	-6.1	40°21′12.36″N	5°41′46.52″W
Canchal Negro	NEG	Bejar	Optimal	2360	26	-7.2	40°20′19.97″N	5°41′22.27″W


We characterized the quality of the habitats of the study area by generating a niche model. We used MAXENT algorithm (Phillips et al., 2006) to generate a model for the Sistema Central according to the species environmental requirements (Morente-López et al., unpublished data). Minimum annual temperature (MAT), precipitation of the driest month (PPd), medium annual snowpack (SP) and medium annual potential evapotranspiration (PET) were used to build the model. We used climatic data from the Atlas Climático de la Península Ibérica with a 200-meter resolution (Ninyerola et al., 2005). Medium annual snowpack was calculated following the methodology proposed by López-Moreno et al. (2007) and the rest of the environmental variables were calculated using ENVIREM R package (Title and Bemmels, 2018). Thus, according to this model, we defined the higher-quality habitats as “Optimal” and the lower-quality habitats as “Marginal” following the definition of environmental marginality (Soule, 1973; Kawecki, 2008). “Optimal” areas were those with habitat suitability values in the highest 33rd percentile of the distribution. The lower values of MAT and PET and the higher values of SP and PPd define these areas. “Marginal” areas were defined as those with habitat suitability values in the lowest 33rd percentile of the distribution. This classification is congruent with demographic trends obtained by Giménez-Benavides et al. (2011a). We selected three *S. ciliata* populations in each of the three mountain ranges, one located in an optimal area and two in marginal areas. The names, location and ecogeographic characterization of the nine studied populations are shown in Table 1.

### DNA Extraction and Molecular Analysis

We collected *S. ciliata* leaf tissue from 20 individuals per population for genetic analysis (*n* = 180). DNeasy Plant minikit (QIAGEN, Valencia, CA, United States) was used for DNA extraction of 10–20 mg of dried *S. ciliata* tissue. Based on a previous study (García-Fernández et al., 2012a), we selected eight microsatellite loci for genotyping: Sci1224, Sci1208, Sci0106, Sci1443, EST-2HTS, EST-37HTS, EST-G34D06 and EST-G47A02. PCR protocols were performed as described in García-Fernández et al. (2012b). We genotyped all samples in an automated DNA sequencer (ABI PRISM 3730, Applied Biosystems, Foster City, CA, United States) in the Unidad de Genómica (Universidad Complutense de Madrid, Spain). GeneMarker version 1.85 (SoftGenetics, State College, PA, United States) was used to determine fragment size. We evaluated genotyping accuracy by re-amplifying and re-scoring 20% of the samples (*N* = 36). MICRO-CHECKER (Van Oosterhout et al., 2004) was used to assess the frequency of null alleles, and allelic dropout. No allelic dropout was found. We detected null alleles in most of the populations, but as they were not related to a particular locus across populations, we kept all the markers. We tested for linkage disequilibrium across loci and populations (Log-likelihood ratio G statistic based on 5000 permutations performed in GENEPOP v. 4.1, Rousset, 2008). Only one locus presented significant linkage disequilibrium across populations. However, we decided to include it in the analysis as the disequilibrium was only present in two of the nine populations.

### Data Analysis

#### Geographic Distribution of Genetic Variation

##### Population genetic characterization

To characterize the study populations, we calculated the following estimators of genetic diversity across populations: (i) total number of alleles (N_a_); (ii) number of private alleles (PA); (iii) mean number of alleles per locus (A); (iv) observed heterozygosity (H_o_); (v) expected heterozygosity (H_e_); and (vi) inbreeding coefficients F_is_ defined as [1-(H_o_/H_e_)] and (v) F_i_, calculated as the probability that the two alleles at a locus are identical by descent following the definition of Malécot and Blaringhem (1948). We applied the methodology proposed by Chybicki and Burczyk (2008) using the Bayesian approach implemented in INEST 2.2 software to calculate H_o_, H_e_ and inbreeding coefficient F_i_ corrected for null alleles. We also tested for deviances from Hardy–Weinberg equilibrium (HWE) per locus in each population following Haldane (1954) and for all populations using Fisher’s exact test. Significance of the latter was assessed with Monte Carlo tests using 2000 iterations. Analyses were performed with *diveRsity* v. 1.9.90 R package (Keenan et al., 2013) implemented in R (R Core Team, 2009).

##### Genetic structure and differentiation among populations

In order to study the contribution to the genetic structuring of the different hierarchical levels in our data set (populations, mountain ranges and environments), we tested for genetic differentiation measured as F_st_ across three different hierarchical configurations: (i) genetic differentiation among and within the nine populations, (ii) genetic differentiation among the three mountain ranges considering the genetic variance among populations within mountain ranges and within populations and (iii) genetic differentiation between environments (optimal *vs.* marginal) considering the genetic variance among populations within environments and within populations (Table 1). F_st_ values and 95% confidence intervals were estimated by 1000 randomizations of bootstrapping distance matrices. We performed the hierarchical genetic structures analysis across these organization levels using *Hierfstats* v. 0.04-22 R package (Goudet and Jombart, 2015). Considering the hierarchical structure of our sampling design, we also performed an analysis of molecular variance (AMOVA) with 9999 permutations, using GeneAlEx 6.5 (Peakall and Smouse, 2012) to contrast the results of the hierarchical F_st_ analysis.

We searched for genetic clusters across spatial scales by performing a Discriminant Analysis of Principal Components (DAPC) (Jombart et al., 2010) as implemented in *adegenet* R package (Jombart, 2008). In DAPC, discriminant functions are linear combinations of the variables (principal components of PCA) which optimize the separation of individuals into pre-defined groups (Jombart and Ahmed, 2012) determined using the K-means clustering algorithm. We used the *find.clusters* function (*adagenet* R package) to study the optimal number of clusters regarding the maximum drop in BIC values. We used the a-score to set the number of PCs retained in the DAPC to control the possible overfit, which is a measure of the trade-off between power of discrimination and over-fitting the model (Jombart and Ahmed, 2012).

We also applied a Bayesian clustering method as implemented in STRUCTURE v 2.3.4 (Pritchard et al., 2000). We performed ten independent runs for each possible number of *K* clusters from one to nine. Each run assumed a burn-in period of 10^6^ iterations, followed by 10^7^ MCMC iterations considering the model of correlation frequencies and admixture origin. To elucidate the most plausible value of *K*, we followed the approach described in Evanno et al. (2005) implemented in Structure Harvester (Earl and vonHoldt, 2012). Then, we used Clumpp v. 1.1.2 (Jakobsson and Rosenberg, 2007) to obtain the permuted membership coefficient of each individual assigned to each cluster, joining the results of the 10 independents runs. The output from Clumpp was visualized by Distruct v 1.1 (Rosenberg, 2004).

A Geneland analysis (Guillot et al., 2005) was also developed considering five runs of 10^6^ MCMC iterations after a burning process of 10^5^ simulations, sampling each 1000 steps, ranging *K* values between 1 and 10 and applying spatial and null alleles corrections, to confirm analysis made with DAPC and STRUCTURE.

#### Geographic and Environmental Drivers of Genetic Structure: Mantel and Partial Mantel Test

We wanted to elucidate whether the observed genetic structure was caused by geographical distance (IBD), environmental conditions (IBE), geographical conformation of the landscape (IBR) or a combination of these factors. To achieve this, we first obtained four different types of distance matrices:

(i) Genetic distance matrix, calculated as pairwise F_st_ distance between the nine populations using FreeNA (Chapuis and Estoup, 2007) to correct for the presence of null alleles; (ii) Geographic distance matrix, based on Euclidean distances between populations. Genetic and Euclidean distance matrices were transformed [F_st_/(1-F_st_)] and log(Euclidean distance), respectively, to linearize their relationship (Rousset, 1997); (iii) The environmental distance matrices were created with 200-meter resolution data from the Atlas Climático de la Península Ibérica (Ninyerola et al., 2005). As annual maximum and minimum temperatures (Tmax and Tmin, respectively) have been proposed as reference variables of environmental gradients in alpine ecosystems (Totland, 1999; Totland and Alatalo, 2002; Körner, 2003, 2007), we selected Tmax and Tmin as proxies of the environmental gradients of the study territory. We previously tested them for collinearity by checking if the variance inflation factor (VIF) was below 2 (Chatterjee and Hadi, 2015); and (iv) Least-cost distance matrix between each pair of populations was calculated in terms of the cost of effective migration from one population to another, using a digital elevation model (DEM) with 200-m spatial resolution (Ninyerola et al., 2005) as a proxy. Taking into account the biological features of this alpine species, effective gene flow was only considered to be possible along the areas of the Sistema Central with the highest elevations. Thus, 1280 m a.s.l. was selected as the threshold below which the species could not migrate. The selected threshold ensures connectivity among the three mountain ranges. Least-cost values were determinate by determined for each pair of populations as the accumulated cost value of all cells to be crossed. Cell cost values express cumulative cost of movement in terms of distance equivalence. Distances were measured according to the minimum amount of friction that must be accumulated to move from one population to another target population. Movements were allowed for the standard eight directions from any cell. Least-cost distances were calculated in IDRISI Selva V. 17 (Eastman, 2012).

Since the adequacy of simple Mantel tests has been considerably criticized in the past for its proneness to type I error (e.g., Manel and Holderegger, 2013) we used reciprocal causal modeling (RCM) to control for Mantel test proneness to spurious correlations (Cushman and Landguth, 2010) and to assess the relevance IBD, IBE and IBR patterns in our study case. We followed the methodology proposed by Cushman et al. (2006, 2013b) and applied by Ruiz-Gonzalez et al. (2015).

Reciprocal causal modeling uses pairs of reciprocal partial Mantel tests to study the relative support of alternative genetic configuration hypotheses (IBD, IBE, IBR). First, the partial Mantel correlation between one of the hypothesis (e.g., IBD) and the genetic distance (G.Dist), controlling for the effect of a second hypothesis (e.g., IBR) was calculated using partial Mantel test (G.Dist ∼ IBD| IBR). Second, a second partial Mantel test was developed but calculating the correlation between the genetic distance and the second hypothesis, controlling for the effect of the first hypothesis (G.Dist ∼ IBR| IBD). The relative support for IBD (focal model) relative to IBR (alternative model) is the difference between the partial correlations of the two tests (IBD| IBR-IBR| IBD) and vice versa (Cushman et al., 2013a,b). Thus, if IBD hypothesis is correct then IBD|IBR – IBR|IBD should be positive and IBR|IBD – IBD|IBR zero or negative, and, conversely, if IBR hypothesis is correct, then IBD|IBR – IBR|IBD should be zero or negative and IBR|IBD – IBD|IBR positive. Following this methodology a full matrix of all the possible hypothesis comparisons was calculated (reciprocal causal modeling matrix). If for a hypothesis all values in a column are positive and all associated values in a row are negative, then that model is fully supported and, thus, such hypothesis is the best compared to all alternatives. For each of the Mantel tests hypothesis combinations we also calculated the correlation values and significance through corrected permutation tests with 9999 permutations. Analyses were carried out using *adegenet* (Jombart and Ahmed, 2012), *ade4* (Dray and Dufour, 2007), *ecodist* (Goslee and Urban, 2007) and *stats* (Pritchard et al., 2000) R packages.

#### Geographic and Environmental Drivers of Genetic Diversity: Spatially Explicit Mixed Models

To study the possible relationship between genetic diversity estimators and geographical and environmental variables, we also developed different geographically explicit generalized linear mixed models (spatial GLMM’s) using genetic, geographical and environmental information. The models were built using spaMM R package (Rousset and Ferdy, 2014). We used different genetic diversity estimators (H_o_, H_e_, F_is_, F_i_, A and PA) as dependent variables and annual Tmax and Tmin as independent variables, considering the geographical coordinates of the locations a random factor. To account for non-linear responses of the environmental variables, we also tested models including their squared values. We tested for normality and homoscedasticity of the model residuals and made the necessary transformations when required. We also tested the possible spatial autocorrelation of the residuals performing a Moran’s I test (Moran, 1950). For each model developed, a *P*-value of an associated likelihood ratio test (LRT) between the “full” model (including environmental variables) and the “null” model (only with spatial random effect) tested the effect of a given factor after applying the Bonferroni correction. We also performed an AIC rank test to select the best fitting model when more than one environmental variable had a significant effect on a genetic diversity estimator and to ensure the improvement of the “full” *versus* the “null” model.

## Results

### Geographic Distribution of Genetic Variation

#### Population Genetic Characterization

The eight microsatellites scored a total of 107 different alleles across all individuals with an average of 5.2 alleles per locus. Number of alleles per population (Na) varied from 38 to 57 and number of alleles per locus from 4.30 to 6.05 (see Table 2). Populations from Guadarrama (MOR, NAJ and PEN) had the lowest number of private alleles (PA) compared to the populations from the other two mountain ranges (Kruskal–Wallis test, *P* = 0.02). Observed heterozygosity values (H_o_) were lower than expected heterozygosity values (H_e_) for all populations except MOR where H_o_ was higher than H_e_. Populations NAJ and SES had similar H_o_ and H_e_ values. All populations except NAJ and PEN (GDM) departed from the H&W equilibrium, and they showed a significant excess of homozygotes across loci (Table 2). Inbreeding coefficients were significantly higher than zero in all populations (*P* < 0.05) except MOR (*F*_is_ = -0.08), NAJ (*F*_is_ = -0.01) and SES (*F*_is_ = -0.01), which were not significantly different from zero (*P* > 0.05 in all cases).

**Table 2:** Estimators of genetic diversity at the population level, fixation indexes, and Hardy-Weinberg exact tests in studied *Silene ciliata* populations.

Pop ID	N	N_a_	A	H_o_	H_e_	P_(HWE)_	F_is_	F_i_	F_i_low_	F_i_high_
NAJ	20	44 (2)	4.86	0.66	0.65	0.083 (0)	-0.01	0.03	0.00	0.08
MOR	20	47 (2)	5.10	0.74	0.68	0.000 (4)	-0.08	0.02	0.00	0.07
PEN	20	38 (0)	4.30	0.48	0.54	0.063 (2)	0.10	0.06	0.02	0.14
SES	20	50 (4)	5.46	0.71	0.70	0.000 (5)	-0.01	0.06	0.04	0.09
CAM	20	48 (3)	5.19	0.57	0.68	0.000 (5)	0.16	0.15	0.00	0.32
ZON	20	41 (3)	4.42	0.65	0.67	0.000 (5)	0.03	0.03	0.00	0.10
RUI	20	55 (5)	5.80	0.60	0.72	0.000 (7)	0.17	0.31	0.29	0.34
AGI	20	57 (7)	6.05	0.54	0.76	0.000 (7)	0.29	0.31	0.16	0.45
NEG	20	53 (5)	5.59	0.45	0.72	0.000 (4)	0.37	0.37	0.18	0.48


*Pop ID, identification of each population as indicated in Table 1; N, number of genotyped samples; N_*a*_, total number of alleles per population with number of private alleles in parentheses; A, mean number of alleles per locus; H_*o*_ and H_*e*_, observed and expected heterozygosity; P_(*HWE*)_, *p*-value from Fisher’s exact test and number of loci out of H&W equilibrium in parentheses (*P* < 0.05); *F*_*Is*_, multilocus fixation index calculated as 1-(H_*o*_/H_*e*_); *F*_*I*_, multilocus fixation index as the probability that two alleles at a locus are identical by descent following the definition of Malécot and Blaringhem (1948).*

#### Genetic Structure and Differentiation Among Populations

When testing for population structure, hierarchical F_st_ analysis showed that genetic differentiation was higher among individuals within populations (F_Ind/Pop_) (Mean [CI]: 0.26 [0.11, 0.41]) than among populations (F_Pop/T_) (0.09 [0.06, 0.15]), although both were significant. The F_st_ hierarchical structure analyses of population nested within mountain ranges showed that all levels contributed to the hierarchical genetic structure with different magnitudes. Mountain range (F_Mt/T_) (0.05 [0.006, 0.11]) and population nested within mountain range (F_Pop/Mt_) (0.06 [0.04, 0.08]) showed a small but significant value. Individuals within populations showed the greatest genetic divergence (F_Ind/Pop/Mt_) (0.26 [0.10, 0.42]). The F_st_ hierarchical structure analyses of population nested within environment found no significant effect of environment (F_Env/T_) (0.0 [-0.02, 0.02]) and a small significant value for population nested in environment (F_Pop/Env_) (0.09 [0.06, 0.15]). As in the F_st_ hierarchical structure analyses, individuals within populations had the greatest genetic divergence values (F_Ind/Pop/Env_) (0.26 [0.11, 0.40]). Results are summarized in Table 3. AMOVA results showed very similar patterns of molecular variance partition (see Supplementary Table S1).

**Table 3:** Hierarchical F_st_ results.

(A) Populations			(B) Mountains and Populations				(C) Environments and Populations			
					
	Pop.	Ind.		Mt.	Pop.	Ind.		Env.	Pop.	Ind.
Total	0.09 (F_Pop/T_)^∗^	0.32 (F_Ind/T_) ^∗^	Total	0.05 (F_Mt/T_)^∗^	0.10 (F_Pop/T_)^∗^	0.33 (F_Ind/T_)^∗^	Total	0.00 (F_env/T_)	0.09 (F_Pop/T_)^∗^	0.32 (F_Ind/T_)^∗^
Pop.	0.00	0.25 (F_Ind/Pop_)^∗^	Mt.	0.00	0.06 (F_Pop/Mt_)^∗^	0.30 (F_Ind/Mt_)^∗^	Env.	0.00	0.09 (F_Pop/Env_)^∗^	0.32 (F_Ind/Env_)^∗^
			Pop.	0.00	0.00	0.25 (F_Ind/Pop/Mt_)^∗^	Pop.	0.00	0.00	0.25 (F_Ind/Pop/Env_)^∗^


*The entire sample was used to analyze the genetic structure based on three hierarchical conformations: ***(A)*** only considering populations, ***(B)*** population nested within mountain range and ***(C)*** population nested within environments. Pop., populations; Mt., mountain ranges; Env., environments; Ind., individuals. We show the *F* values for each of the conformations represented in parentheses. For example, F_*Mt/T*_ represents the genetic variance among mountains, F_*Pop/Mt*_ genetic variation among populations within mountains and F_*Ind/Pop/Mt*_ genetic variation among individuals within populations within mountains. Asterisks denote significant values tested by bootstrapping with 1000 randomizations.*

**FIGURE 2:**
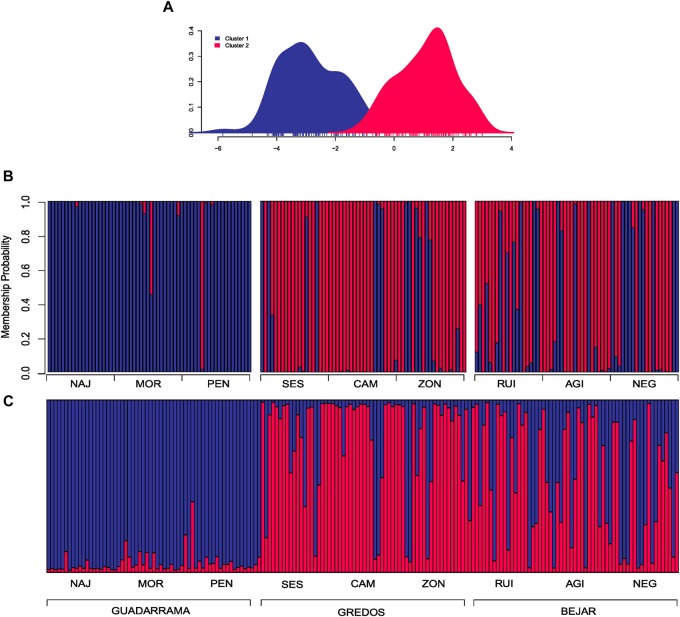
Discriminate analysis of principal components (DAPC) and Bayesian analysis of population structure (STRUCTURE) for two clusters conformation (*K* = 2). **(A)** Scatterplot of the first two principal components showing the differentiation between the two groups by colors. **(B)** DAPC composition plot (compoplot) of each individual grouped by mountain ranges. Colors represent the same clusters as the scatterplot. **(C)** STRUCTURE composition plot (compoplot) of each individual grouped by mountain ranges.

**FIGURE 3:**
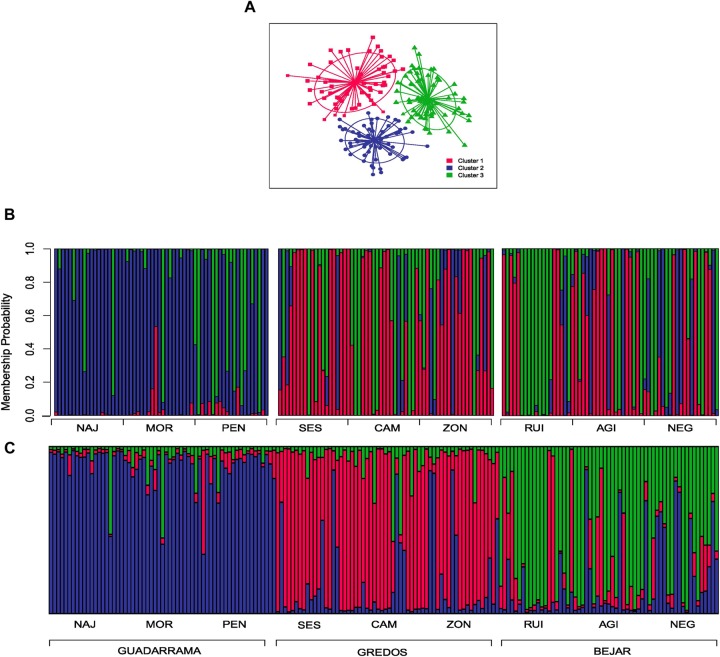
Discriminate analysis of principal components (DAPC) and Bayesian analysis of population structure (STRUCTURE) for *K* = 3 clusters. **(A)** Scatter plot of the first two principal components showing the differentiation between the three groups by colors and inertia ellipses. Dots, squares and triangles represent individuals of each cluster. **(B)** DAPC composition plot (compoplot) of each individual grouped by mountain ranges. Colors represent the same clusters as the scatterplot **(C)** STRUCTURE composition plot (compoplot) of each individual grouped by mountain ranges.

In the DAPC clustering analysis, we selected *K* = 2 and *K* = 3 structures based on the BIC curve which represents the plausible number of clusters in the data (Supplementary Figure S1) and the biological meaning of our study case. In the *K* = 2 partition, individuals were neatly classified into two groups; one mainly composed of individuals from the GDM mountain range and the other of individuals from GRD and BJR (Figures 2A,B). *K* = 3 genetic data partition showed one group mostly composed of individuals from the three populations of the GDM mountain range. The other two groups were essentially a mixture of individuals from populations of the GRD and BJR mountain ranges (Figures 3A,B).

STRUCTURE showed that *K* = 2 best explained the genetic structure of the study data set (Supplementary Figure S2), separating individuals from the GDM mountain range and individuals from the GRD and BJR mountain ranges (Figure 2C). These results agree with those obtained in DAPC. The second most plausible structuring of the data was *K* = 3, with one group corresponding to each mountain range (GDM, BJR and GRD) (Figure 3C). Geneland results were consistent with the genetic structure founded with DAPC and STRUCTURE analysis (Supplementary Figure S3).

### Geographic and Environmental Drivers of Genetic Structure: IBD, IBR and IBE

IDB arises as the strongest overall hypothesis regarding to the relative support values of the RCM matrix (Table 4A), presenting positive values in the entire column and negative values in the entire row. In a similar way, all partial Mantel tests values relates with this variable showed a significant and positive correlation between genetic and Euclidian distance (Table 4B). Regarding to the IBR hypothesis, negative relative support value arises when it was controlled by IBD hypothesis, but positive values emerge when it was controlled by IBE hypotheses (Table 4A). Weak and no significant correlation was found when partial Mantel test related with this variable was controlled by the Euclidean distance (Table 4B). Both IBE hypotheses are the ones with less support regarding to the RCM matrix and with none significant Mantel correlation values.

**Table 4:** Reciprocal causal modeling results.

		IBD	IBR	IBE	
				
		Eu.Dist.	Cost.Dist.	Tmax.Dist.	Tmin.Dist.
**(A) Reciprocal causal modeling matrix**					
IBD	Eu.Dist.	**0**	-0.4	-0.7	-0.5
IBR	Cost.Dist.	**0.4**	0	-0.6	-0.4
IBE	Tmax.Dist	**0.7**	**0.6**	0	-0.2
	Tmin.Dist	**0.5**	**0.4**	0.2	0
**(B) Mantel correlation models matrix**					
IBD	Eu.Dist.	**0.7^∗∗^**	0.06	0.02	0.2
IBR	Cost.Dist.	**0.4^∗∗^**	**0.6^∗∗^**	-0.03	0.2
IBE	Tmax.Dist	**0.7^∗∗^**	**0.6^∗^**	0.1	0.09
	Tmin.Dist	**0.7^∗∗^**	**0.6^∗∗^**	0.3	0.1
^∗∗^ < 0.002, ^∗^ < 0.01					


***(A)** Reciprocal causal modeling matrix for IBD, IBR and two IBE hypothesis. Columns indicate focal models (e.g., IBD|IBR), and rows indicate alternative models (e.g., IBR|IBD). Each value represents the relative support of the focal model (e.g., IBD|IBR – IBR|IBD). **(B)** Partial Mantel correlation matrix summarizes the *r* values of the model defined with the column variable controlling by the row variable. The diagonal values are the simple Mantel test *r* of a variable. IBD, isolation by distance; IBR, isolation by resistance; IBE, isolation by environment; Eu.Dist., Euclidean distance; Cost.Dist., cost distance; Tmax.Dist., environmental distance related with annual maximal temperature; Tmin.Dist., environmental distance related with annual minimum temperature. Bold values represents positive correlation values (section A) and significant correlations (section B).*

### Geographic and Environmental Drivers of Genetic Diversity

**FIGURE 4:**
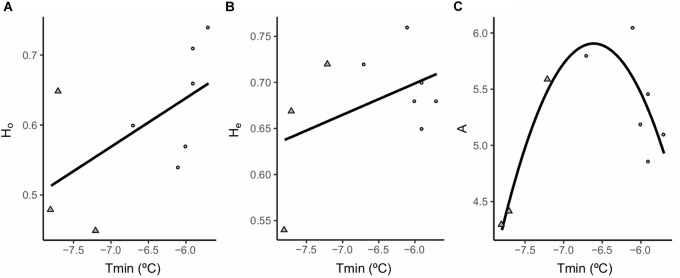
Significant relationships between the different genetic diversity estimators and the environmental variables used in the models. **(A)** Ho, observed heterozygosity; **(B)** He, expected heterozygosity; **(C)** A, mean allelic richness per locus (average number of alleles per locus). Tmin; minimum annual temperature. Dots represent populations classified as “marginal” and triangles populations classified as “optimal.”

Spatially explicit mixed models found a positive linear relationship between annual minimum temperatures (Tmin) and Ho (β = 0.009, df = 4.3, *P* < 0.05) and He (β = 0.004, df = 6.3, *p* < 0.05) and a quadratic relationship between Tmin and mean number of alleles per locus (A) (β = -0.0003, df = 4.6, *P* < 0.001) (Figure 4 and Table 5). Notably, intermediate levels of Tmin exhibited the highest levels of A. Maximum annual temperature (Tmax) was not related to Ho, He or A. The addition of Tmin as an explanatory variable significantly improved AIC values in the models compared to the “null” models (Table 5). No relationship was found between any of the environmental variables and Fi, Fis or PA. No significant spatial autocorrelation of the residuals was found.

**Table 5:** Mixed effect models fitted to test the effect of environmental factors (fixed factors) in determining different estimates of genetic diversity across populations.

Response variable	Tested model	*P*-value Bonferroni (LRT)	β [CI]	Lambda ()	DF	mAIC
**H_o_**						
Model1	H_o_ ∼ Tmin + (1|longitude + latitude)	<0.05	0.009 [0.00004, 0.02]	0.004	4.3	-8.8
Null Model	H_o_ ∼ 1 + (1|longitude + latitude)					-6.9
**H_e_**						
Model2	H_e_ ∼ Tmin + (1|longitude + latitude)	<0.05	0.004 [0.001, 0.07]	0.002	6.1	-26.1
Null Model	H_e_ ∼ 1 + (1|longitude + latitude)					-16.5
**A**						
Model3	A ∼ Tmin^2^ + (1|longitude + latitude)	<0.001	-0.0003 [-0.0004, -0.0002]	0.24	4.6	5.6
Null Model	A ∼ 1 + (1|longitude + latitude)					19.7


*The spatial location of each population is accommodated as a random factor. For each model the *P*-values of an associated likelihood ratio test (LRT) analyze the effect of a given factor after applying the Bonferroni correction. H_*o*_, observed heterozygosity; H_*e*_, expected heterozygosity; A, mean allelic richness per locus (average number of alleles per locus); Tmin, annual minimum temperature; β, model estimator; CI, confidence interval; DF, degrees of freedom; lambda, dispersion parameter; mAIC, Akaike information criteria value.*

## Discussion

Results depict a complex scenario in which geographical and environmental factors influence the genetic structure and diversity of *S. ciliata* in the Central System of the Iberian Peninsula. *S. ciliata* presented a marked genetic structure, and IBD was the main factor shaping genetic patterns. No genetic structure was found between populations from optimal and marginal habitats suggesting no isolation effects related to environmental differences. However, a significant relationship was found between environmental variables and genetic diversity.

### Geographic Distribution of Genetic Variation

Hierarchical analyses showed that most of the genetic diversity resided within populations. This is probably due to the species’ breeding system which favors allogamous crossings (Giménez-Benavides et al., 2007b). It also reflects the presence of significant within-population genetic structure due to limited gene flow (Lara-Romero et al., 2016b), which is shaped by limited effective pollen and seed dispersal distances (Lara-Romero et al., 2014, 2016a,b). A significant genetic variation component between populations within mountain ranges was also observed, but no genetic differentiation was found when we contrasted populations inhabiting optimal and marginal habitats across mountain ranges. This suggests that genetic differentiation between populations within mountain ranges is found between marginal habitats. Hierarchical F_st_ found significant differentiation between mountain ranges of similar magnitude to that between populations within mountain ranges, which was further supported by clustering analyses. GDM was more differentiated than the other two mountain ranges (BEJ and GRE) as shown by the *K* = 2 conformation in the clustering analysis. On the other hand, the *K* = 3 conformation was clearly related to the three mountain ranges in STRUCTURE, and with an admixture between BEJ and GRE in DAPC. These results show the relevance of the spatial configuration of the landscape at the mountain range level on the genetic structure. This could be related with the colonization pattern of the species along the East-West oriented axis of the Sistema Central (see Figure 1). Patterns of genetic variation across landscapes in alpine ecosystems are diverse and context dependent. Geng et al. (2009) found short-distance genetic structure patterns related to limited gene dispersal along with substantial levels of gene flow and slow rates of genetic drift between topographically separated populations. Many other authors reported genetic structure at larger landscape scales (e.g., Aægisdóttir et al., 2009; Lega et al., 2014) related to natural barriers to gene flow. In our case, we detected both coarse-scale mountain range clustering and finer-scale short distance population differentiation. Our results suggest a prevalence of gene flow limitation between mountain ranges, with considerable genetic differentiation among populations and mountain ranges.

### Geographic and Environmental Drivers on Genetic Structure: IBD, IBR, and IBE

The significant relationship between geographical distance and genetic differentiation based on RCM and Mantel correlations suggested an IBD pattern between *S. ciliata* populations. IBD is the most common pattern of genetic differentiation in landscape genetics studies (Jenkins et al., 2010; Cushman et al., 2015), including alpine plants (e.g., Stöcklin et al., 2009; Wu et al., 2015). Nevertheless, the IBD hypothesis cannot be generalized in alpine ecosystems (Frei et al., 2012a,b). When we considered the topography of the study territory, we also found an IBR pattern showing a significant effect of cost distances in the genetic differentiation, although weaker than the IBD. This is not surprising since the IBR calculated based on the DEM includes a distance effect. The East-West alignment of the mountain ranges which conforms the Sistema Central (see Figure 1) includes passages of lower elevation that connect the mountain ranges and act as topographic barriers. This effect works in the same direction as the isolation effect imposed by the Euclidean distance (IBD). Thus, the East-West orientation of the mountain ranges and the associated barriers seem to be important drivers of the genetic structure at the landscape scale in our study.

No genetic structure associated to environmental differentiation was detected using RCM and partial Mantel correlations, or the genetic structure clustering and hierarchical F_st_ analyses approaches. Previous *S. ciliata* field studies carried out in similar areas found evidence of local adaptation in marginal populations, i.e., seed germination success and survival compared to optimal populations (Giménez-Benavides et al., 2007a,b). They also inferred a putative genetic isolation pattern between optimal and marginal populations because of mismatched flowering periods (Giménez-Benavides et al., 2007b, 2011b), measured under field conditions. Restriction of gene flow due to phenological mismatches, and possible differential selection along gradients, may cause genetic differentiation (Hirao and Kudo, 2004). However, a subsequent genetic study carried out in GDM populations (García-Fernández et al., 2012b) found significant gene flow along an elevational gradient and low genetic differentiation. Using AFPLs, Manel et al. (2012) showed that environmental variables are drivers of plant adaptation at the scale of a whole biome for a large number of alpine species. Furthermore, several studies in mountain ecosystems have found adaptive variation patterns using genomic approaches (e.g., Poncet et al., 2010; Mosca et al., 2012, 2014; Di Pierro et al., 2017). These previous results in our and other alpine species put us on the track of adaptive divergence between populations. The lack of an environmental signature in the genetic differentiation found in our study may result from the low number of neutral molecular markers used and does not preclude environmentally induced genetic signals in other areas of the *S. ciliata* genome. IBE patterns can be detected using neutral markers because the signature of selection extends to genome areas beyond the genes that are under selection (Shafer and Wolf, 2013; Sexton et al., 2014; Kern and Hahn, 2018), but they are certainly more difficult to find when the number of loci used is low. Further research using genomic and quantitative genetic approaches is needed in Mediterranean alpine ecosystems to provide insight into the identification of genetic differentiation patterns related to adaptation along environmental gradients.

### Geographic and Environmental Drivers on Genetic Diversity

In contrast with the lack of an environmental signature on genetic differentiation, we found a significant relationship between environmental variables and genetic diversity. Under an ecological niche perspective of the central-marginal model, optimal environmental conditions in the central areas allow larger and more stable populations with greater genetic diversity (Kawecki, 2008; Sexton et al., 2009). Conversely, populations in marginal habitats tend to be small and fluctuating in size and, therefore, prone to suffer bottleneck processes that causes genetic diversity erosion (Glémin et al., 2003; Whitlock, 2004; Kawecki, 2008). Consequently, genetic diversity is expected to be higher in optimal populations (Eckert et al., 2008; Pironon et al., 2015).

Contrary to our expectations, the lower Tmin values which entail optimal conditions for the species (Giménez-Benavides et al., 2007a, 2011a; Lara-Romero et al., 2014) were related to lower genetic diversity values. This apparent contradiction may be partially explained by a historical signature of genetic diversity patterns that overrides the effects of current conditions. The effects of glacial cycles have often been used to explain distributional shifts of species, as well as the contraction, fragmentation and connectivity of mountain populations (Marques et al., 2016). Thus, glacial pulses would have been responsible for shifting *S. ciliata* populations to lower elevations, allowing populations from nearby mountains to be connected (Hewitt, 2001; García-Fernández et al., 2013). As a result, these lower areas would have increased their genetic diversity and acted as a genetic reservoir for the species (Knowles, 2001; Tzedakis et al., 2002; Holderegger and Thiel-Egenter, 2009). Postglacial recolonization of higher altitudes from lower areas (Dechaine and Martin, 2004) would have originated a front advancing population edge by a subsample of the population with lower genetic diversity.

H_o_ and H_e_ values were the highest in the marginal populations at the lowest elevations, but allelic richness decreased sharply compared to populations at intermediate elevations and lower annual minimum temperatures fitting a quadratic response. The low population size and possible bottlenecks experienced by the most marginal populations, potentially associated with current high annual minimum temperatures may be responsible for this. This mismatch between allelic richness and heterozygosity values could be related to the impact of population bottlenecks on allelic diversity which is often greater and faster than on heterozygosity (Frankham et al., 2002).

We, thus, suggest that the observed genetic diversity pattern is a combination of present and past climatic factors and events. Present marginal conditions associated with lower elevations resulting from altitudinal shifts and climate warming may overlay a genetic diversity pattern that stems from the glacial pulses of the past involving both environmental and geographic factors. In the central-marginal model context, associated genetic diversity patterns can be highly context and scale dependent (Hampe and Petit, 2005; Hardie and Hutchings, 2010). Geographical, ecological and historical gradients act in conjunction creating diverse patterns that cannot be homogenized under a common rule (Sagarin and Gaines, 2002; Eckert et al., 2008; Duncan et al., 2015; Pironon et al., 2017).

## Conclusion

Our results highlight the complexity of the patterns shaping the spatial distribution of genetic variation of plants inhabiting high mountain ecosystems. IBD arises as the main pattern shaping genetic structure in mountain ecosystems. In addition, IBR emerged as another important pattern shaping genetic structure although weaker than IBD when geographical distance and barriers works in the same direction. IBE should be considered as an important force in shaping genetic variation, especially in steep environmental ecosystems like mountainous regions. Present and past changes in environmental conditions inside distribution ranges strongly affect genetic diversity in alpine species.

The results of this study can be useful in a future comparison using populations inhabiting similar environmental gradients in other areas of the species distribution not represented here. This would warrant a more thorough perspective of the main drivers shaping plant populations genetics in widespread Mediterranean Alpine plants. Additional research using genomics and quantitative genetics arise as the path to further understand the variation patterns linked to Mediterranean alpine environments.

## Data Availability Statement

The dataset generated and used in this publication can be found in the Universidad Rey Juan Carlos data repository e-cienciaDatos (https://edatos.consorciomadrono.es/dataset.xhtml?persistentId=doi:10.21950/GSTZ26) doi: 10.21950/GSTZ26.

## Author Contributions

JM-L, AG-F, CL-R, and JI designed this study. JM-L and AG-F collected the samples and performed the laboratory work. JM-L, CG, AG-F, and DD analyzed the data. JM-L wrote the paper with the help of CG, CL-R, and JI. All authors reviewed the paper and approved the final manuscript.

## Conflict of Interest Statement

The authors declare that the research was conducted in the absence of any commercial or financial relationships that could be construed as a potential conflict of interest.
