# Supplementary material for: Geography and Environment Shape Landscape Genetics of Mediterranean Alpine Species Silene ciliata Poiret. (Caryophyllaceae)
Source: Front Plant Sci. 2018 Nov 27;9:1698. doi: 10.3389/fpls.2018.01698 (PMC6277476; doi:10.3389/fpls.2018.01698)
Supplement: Supplementary file 1 [file Table_1.DOCX]

Supplementary Material

Geography and environment shapes landscape genetics of Mediterranean alpine species *Silene ciliata* Poiret. (Caryophyllaceae).

Javier Morente-López^*^, Cristina García, Carlos Lara-Romero, Alfredo García-Fernández, David Draper, José María Iriondo.

*** Correspondence:**Javier Morente-López: javimorente@gmail.com

# Supplementary Figures

# Supplementary Figure 1. Changes in Bayesian Information Criterion (BIC) values in successive increasing of K-means clustering (Jombart and Ahmed, 2012).

# Supplementary Figure 2. ΔK rate of change in the probability between successive runs, as a function of K (Evanno et al. 2005).

# Supplementary Figure 3. Geneland analysis results. (A) Markov Chain Monte-Carlo (MCMC) behavior and number of clusters founded after a burnin of 100x1000 iterations. (B) K=2 composition plot (compoplot) of each individual grouped by mountain ranges and by populations inside mountain ranges. (C) K=3 compoplot of each individual grouped by mountain ranges. Notice that results agree with the DAPC and STRUCTURE analysis.

# Supplementary Tables

# Supplementary Table 1. Molecular variance analysis (AMOVA) of *Silene ciliata* populations in the Central System of the Iberian Peninsula. Pops.: genetic differentiation within and among populations; Mt. & Pops.: among mountains among populations within mountains and within populations; Env. & Pops.: between environments, among populations within environments and within populations; df: degrees of freedom; Est. Var.: variance estimator; %: percentage of variance.

|  | **AMOVA results** | | | | | |
| --- | --- | --- | --- | --- | --- | --- |
| **1. Pops.** |  | df | SS | MS | Est. Var. | % |
|  | **Among Pops** | 8 | 111,20 | 13,90 | 0,28 | 10% |
|  | **Within Pops** | 351 | 903,67 | 2,57 | 2,57 | 90% |
|  | **Total** | 359 | 1014,87 |  | 2,85 | 100% |
| **2. Mt. & Pops.** |  | df | SS | MS | Est. Var. | % |
|  | **Among Mountains** | 2 | 53,51 | 26,75 | 0,14 | 5% |
|  | **Among Pops** | 6 | 57,68 | 9,61 | 0,17 | 6% |
|  | **Within Pops** | 351 | 903,67 | 2,57 | 2,57 | 89% |
|  | **Total** | 359 | 1014,87 |  | 2,89 | 100% |
| **3. Env. & Pops.** |  | **df** | **SS** | **MS** | **Est. Var.** | **%** |
|  | **Among Environments** | 1 | 12,72 | 12,72 | 0,00 | 0% |
|  | **Among Pops** | 7 | 98,47 | 14,06 | 0,28 | 10% |
|  | **Within Pops** | 351 | 903,67 | 2,57 | 2,57 | 90% |
|  | **Total** | 359 | 1014,87 |  | 2,86 | 100% |
