# Supplementary figures and images for: Geography and Environment Shape Landscape Genetics of Mediterranean Alpine Species Silene ciliata Poiret. (Caryophyllaceae)
Source: Front Plant Sci. 2018 Nov 27;9:1698. doi: 10.3389/fpls.2018.01698 (PMC6277476; doi:10.3389/fpls.2018.01698)

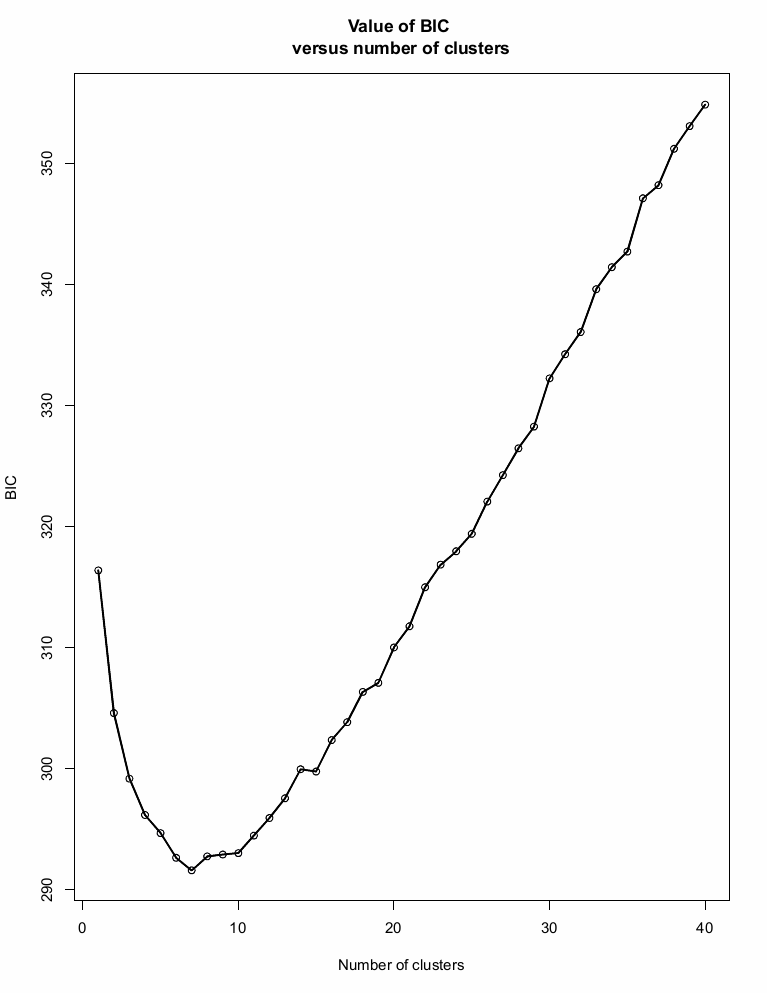

Supplement: Supplementary file 2 [file Image_1.TIF]

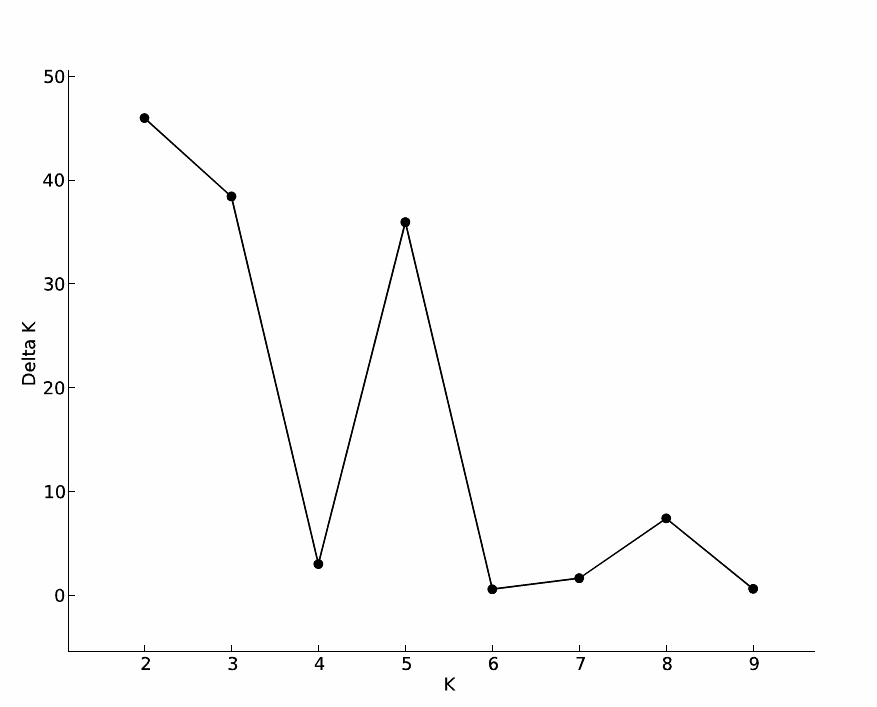

Supplement: Supplementary file 3 [file Image_2.TIF]

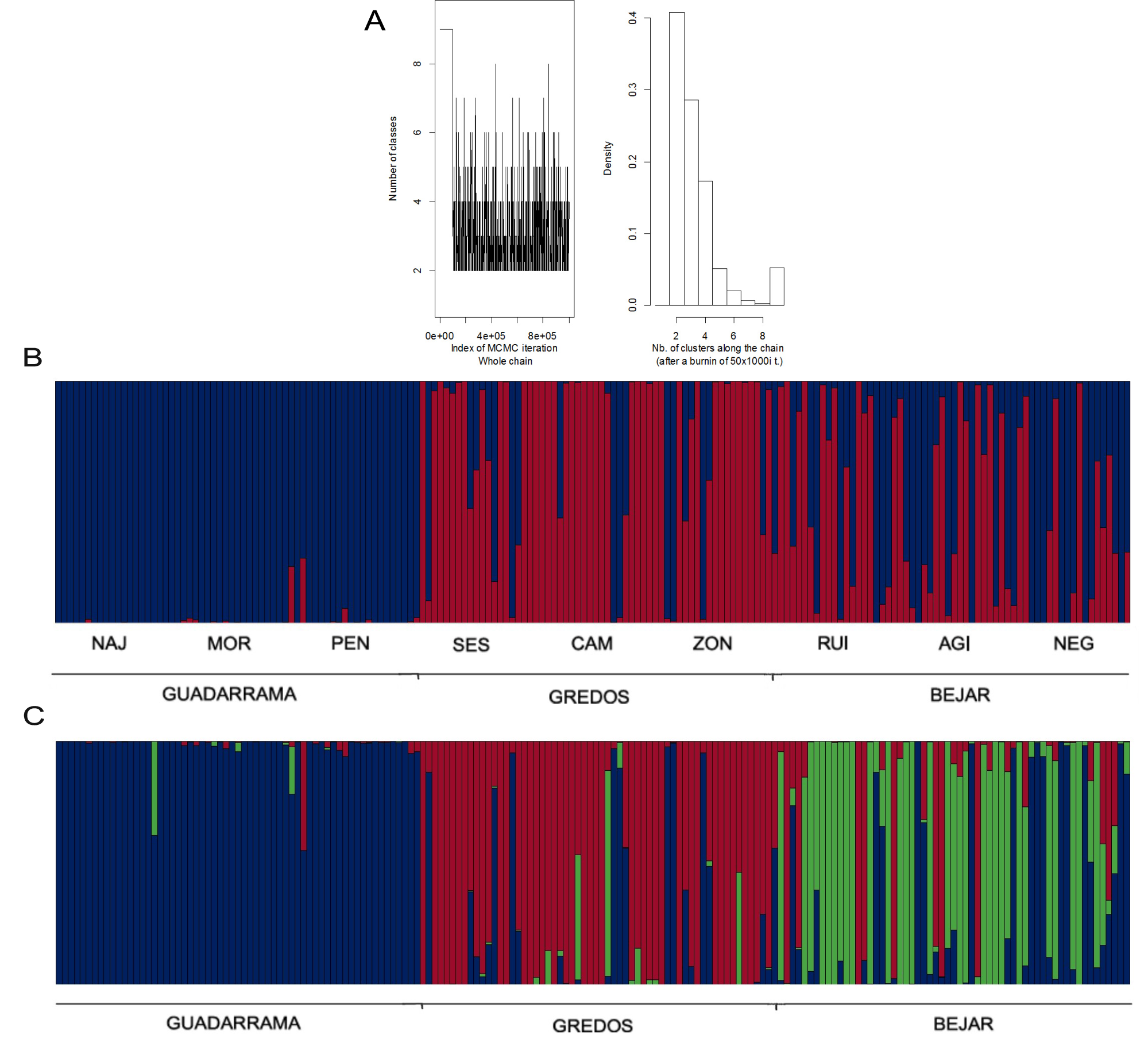

Supplement: Supplementary file 4 [file Image_3.TIFF]
